# Development and Validation of an Algorithm to Objectively Quantify Adherence to Offloading Interventions

**DOI:** 10.1002/jfa2.70170

**Published:** 2026-06-03

**Authors:** Sai V. Yalla, Sanam N. Jhaveri, Nahir S. Negron‐Fernandez, Ryan T. Crews, Noah J. Rosenblatt

**Affiliations:** ^1^ Center for Lower Extremity Ambulatory Research (CLEAR) Dr. William M. Scholl College of Podiatric Medicine at Rosalind Franklin University of Medicine and Science North Chicago Illinois USA; ^2^ Department of Podiatric Medicine & Surgery Rosalind Franklin University of Medicine & Science North Chicago Illinois USA

**Keywords:** accelerometer, cast walker, diabetes, foot ulcer, standing time, step count

## Abstract

**Background:**

Diabetic foot ulcers require offloading for healing. However, adherence with removable offloading devices is often poor. Prior objective measures of adherence yielded important behavioral insights, despite substantial methodological limitations. This study sought to address these limitations by developing a method to track offloading adherence during walking and standing paired with tracking contralateral therapeutic footwear (shoe‐lift) use.

**Methods:**

Thirty healthy adults wore a removable cast walker (RCW) and contralateral shoe‐lift. Accelerometers were placed on both and unilaterally on participants' thighs. Participants performed activities in the laboratory that were logged by investigators, after which monitoring continued for 24 h with participants logging footwear use in a diary. Walking and standing episodes were quantified via the thigh worn monitor. A custom algorithm was developed to classify RCW and shoe‐lift adherence during walking and standing based on sample‐to‐sample variance thresholds. Algorithm accuracy was determined by comparing its adherence metrics with log entries.

**Results:**

The final algorithm resulted in > 99% accuracy in detecting steps and standing while using the RCW and shoe‐lift during in‐lab testing. In 24‐h, community monitoring, algorithm‐derived adherence showed excellent agreement with diary‐based adherence (ICC > 0.96, *p* < 0.01). Error rates (misclassifications) were < 3%.

**Conclusion:**

A method to objectively quantify RCW and contralateral shoe‐lift adherence use was developed and validated. Strong agreement with lab observations and user diaries support its use for monitoring real‐world offloading behavior. This method will allow for enhanced assessments of adherence in future trials and may eventually be incorporated into clinical practice for patient monitoring.

## 1 Introduction

Diabetes‐related foot disease, which includes “peripheral neuropathy, peripheral artery disease, infection, ulcer(s), neuro‐osteoarthropathy, gangrene, or amputation” [1], is one of the most costly complications of diabetes, primarily due to the challenges of preventing and treating diabetic foot ulcers (DFU) [2, 3, 4]. These ulcers often arise from physical activities that impart excessive stress to the feet. Ineffectively managed DFUs may become chronic and necessitate further interventions, including lower extremity amputations [5, 6, 7]. A key approach to managing DFUs involves “offloading”—removing pressure (loads) from the DFU location and other at‐risk areas of the foot via specialized footwear [8, 9].

The gold‐standard in offloading is the knee‐high, nonremovable device, for example, the total contact cast [8, 10]. However, these devices require increased time and skill to apply relative to devices that can be removed, can restrict access to the DFU site, and are generally less preferred by patients [10, 11]. As such, clinicians more frequently rely on removable offloading options [11, 12], with the removable cast walker (RCW) being one of the most commonly employed. While RCWs offer similar plantar pressure reductions as the total contact cast [13, 14], they have yielded poorer healing outcomes [14, 15], attributable to the patients ability to doff the RCW as desired and engage in weight‐bearing activity without protection (i.e., nonadherence) [8]. In fact, patients tend to wear their RCW for only 28%–60% of their daily steps [16, 17, 18], with lower adherence strongly predicting worse healing outcomes [17].

Before low adherence can be managed, better measurement is needed. Self‐reporting of adherence is not reliable, reflecting recall error, and/or deliberate misreporting [19, 20, 21]. While dual accelerometer methods have been used to objectively evaluate adherence to RCWs [16, 17, 18, 22], several limitations exist. First, offloading adherence (OA) has traditionally been assessed only during ambulation [16, 18, 22], without considering standing. This is not trivial, as most weight‐bearing time in patients with, and at‐risk for, DFUs occurs while standing [23, 24]. Moreover, a comprehensive understanding of OA may require accounting for contralateral footwear in addition to the RCW. Current international guidelines recommend that providers consider concurrent use of a contralateral shoe lift to mitigate RCW‐induced limb length discrepancies that can negatively impact users' comfort and gait [8, 25]. However, prior monitoring has not evaluated lift adherence, limiting full insight into offloading behavior.

The purpose of this study was to validate a method for objectively monitoring OA using accelerometry based activity monitors that can account for walking and standing, and for use of a contralateral shoe lift during these activities. Such methods could be used to better guide treatment plans for non‐healing DFUs by understanding the extent to which poor healing reflects patient‐specific behavior, or unsuccessful medical interventions targeting the wound itself.

## 2 Methods

### 2.1 Subject Demographics

We recruited 30 individuals (age: 25.8 ± 4.3 yrs; height: 1.70 ± 0.08 m; weight: 72.5 ± 16.9 kg; 13 M/17F) to participate in this study that was approved by the Rosalind Franklin University IRB. Participants had to be 18 years or older with no lower extremity injuries in the past 6 months and able to walk without any assistive device. Individuals with peripheral neuropathy, an active DFU, or extensive experience wearing an RCW were excluded. Following screening, we collected subject demographics and shoe size. Participants were randomized using a predetermined block randomization sequence to receive either a knee‐high RCW (DH Walker, Össur, Reykjavik, Iceland) or an ankle‐high RCW (low top Equalizer Walker Össur, Reykjavik, Iceland) to be worn on either the right or left limb. In addition, a study‐provided shoe lift (EVENup, OPED, Braselton, GA, USA) was placed on a standardized athletic shoe worn by all participants. Participants accommodated to the devices before data was collected.

### 2.2 Instrumentation

**FIGURE 1:**
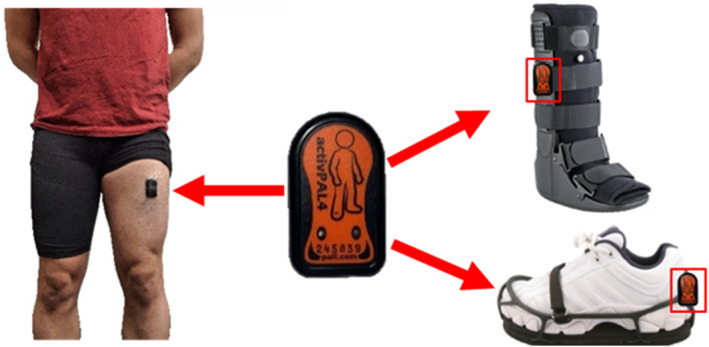
ActivPal activity monitor locations. A monitor (middle) placed on the participant's thigh (left) was synced to monitors placed on the RCW (top right) and on the contralateral lift, which was attached to a study‐provided shoe (bottom right). The thigh monitor served as the gold standard for identifying activity (walking or standing). Data from the device monitors, along with the gold standard, was entered into a custom algorithm to identify the amount of activity that was performed while concurrently using the devices.

Activity data was collected using three activPAL4 (PAL Technologies Ltd, Glasgow, Scotland) miniature electronic monitors. The monitors were wrapped in a waterproof nitrile covering before being placed on the body or device. One monitor was placed on the middle of the quadricep of the thigh on the side with the RCW, following manufacturer instructions (Figure 1). This placement ensures that data can be processed via the PAL technologies custom VANE algorithm to accurately identify sitting, standing and stepping [26]. The thigh monitor was adhered to the thigh with a waterproof 13 × 13 cm sheet of 3M Tegaderm dressing. A second activPal monitor was placed on the lateral side of the RCW in between the soft liner and the rigid strut (Figure 1). Finally, a third monitor was placed behind the heel portion of the shoe lift (Figure 1). All monitors were synchronized in the PAL software to simultaneously start recording.

### 2.3 In‐Lab Testing

After being fitted with devices, all participants performed a series of activities of daily living in the laboratory (Table 1). All tasks were initially performed without the RCW or the contralateral lift (*device‐off* condition), using standardized athletic shoes, and then repeated while wearing them (*device‐on* condition). A single observer recorded the start and end times of each activity and the number of steps taken during each.

**TABLE 1:** In‐lab activities performed with and without devices.

Activities	Description
Walking	Participants walked back and forth within a 35‐m hallway at three walking speeds (habitual, fast and slow)
Sitting	Participants sat quietly in a chair with their feet planted flat on the ground for 1‐min.
Bending	Participants bent down, picked up an object from the floor, stood completely straight up with the object in hand, and then placed it back down on the floor. The task was performed twice with specific instructions as to how to move each time, i.e. bend from waist or flex knees.
Laying down	Participants laid quietly on their back on the ground or a medical table for 1‐min.

### 2.4 Community Monitoring

For the 24‐h period following completion of laboratory activities, participants were instructed to alternate (at the timing of their choice) between wearing the RCW alone, the lift alone, or both. Each participant was also given a diary and instructed to record the times when they donned and doffed their RCW and/or lift. After 24 h, participants visited the lab to return the monitors and diary.

### 2.5 Development and Validation of Adherence Algorithm

After monitors were returned, raw accelerometer data were exported and processed using the PAL analysis software's VANE algorithm. The software provided step counts, walking time and upright time (standing + walking) during 15 s epochs. Standing time was computed as upright time minus walking time. As the activPAL sensors have only been validated for quantifying physical activities when positioned on a user's thigh, which permits assumptions for determining whether the person is upright based upon the sensors orientation and rotation relative to its *y*‐axis, it was assumed that adherence with the RCW or shoe‐lift could not be determined by simply time synchronizing activPAL processed data from devices with that of the thigh sensor.

**FIGURE 2:**
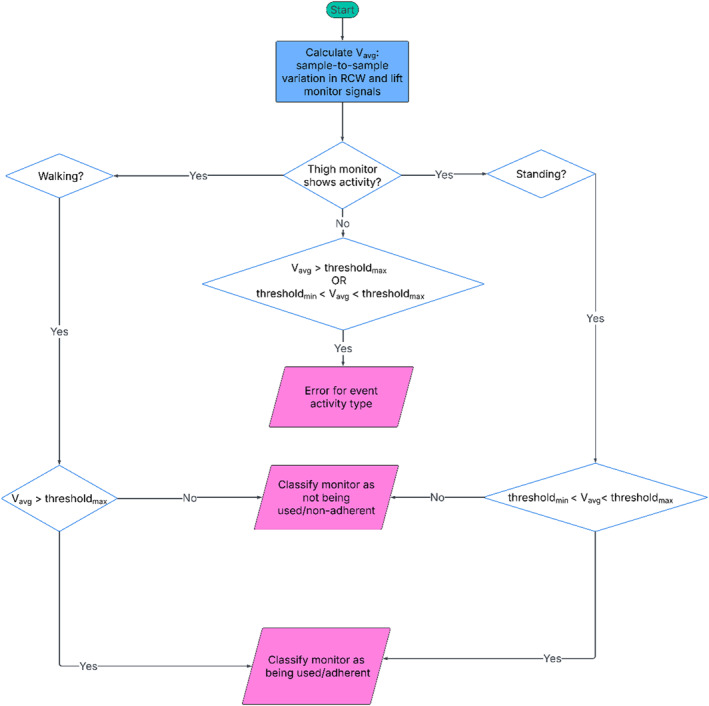
Overview of the custom algorithm. For each epoch, average sample‐to‐sample variance (*V*_avg_) is calculated from a given device monitor that has been processed through VANE. For those epochs in which walking or standing are identified on the thigh monitor, *V*_avg_ is compared to established thresholds. If *V*_avg_ falls within threshold boundaries, then the algorithm indicates that the device is being used while the activity is occurring. From this classification, we determined accuracy during the laboratory activities or adherence during functional testing in the community. The initial development stage followed this same process with the addition that the thresholds were iteratively adjusted to optimize accuracy.

Processed data was separated into the in‐lab activity data and the 24‐h community recordings based on time stamps and recorded stops and starts. Processed data from the in‐lab activities was used to develop a custom‐built adherence algorithm (LabVIEW, National Instruments, Ausin, TX). The algorithm was intended to identify standing and walking periods, based on the thigh‐worn sensor, that occurred concurrently with use of the RCW and/or lift (Figure 2). During development, the goal was to maximize accuracy, that is output a high percentage of device use during walking and standing in the *device‐on* condition and a low percentage in the *device‐off* condition.

Using data from the device monitors of five randomly selected participants, we calculated the mean sample‐to‐sample variation across the X, Y, and Z axes for each epoch as *V*_avg_ = (X + Y + Z)/3, where X, Y, and Z are directly provided by VANE. We reasoned that high variations would well distinguish walking and standing, that is high variation would be indicative of walking and low variation would indicate standing, with zero variance indicating stationarity (non‐use). For each of the selected subjects, we identified the maximum and minimum V_avg_ in RCW and lift monitors during thigh‐monitor‐defined walking and thigh‐monitor‐defined standing. The extremes of these values across subjects defined initial estimates of thresholds that could potentially distinguish standing and walking activity (threshold_min_ and threshold_max_). These initial thresholds served as starting points used in optimization routines to achieve the development goal.

Specifically, for each epoch of thigh‐defined standing or walking, the RCW and lift were first classified as being used for that activity if threshold_min_ < *V*_avg_ < threshold_max_ or if V_avg_ > threshold_max,_ respectively. For given thresholds, the accuracy of identifying device usage during an activity was noted. Both thresholds were then incrementally varied over ranges based on the variances in subject‐specific maxima and minima for V_avg_, to determine the threshold values that maximized accuracy. The process was done separately for the RCW and for the lift.

The optimized thresholds, which were identical for the RCW and lift monitors, were subsequently applied to all subjects to quantify walking and standing activity performed while using the RCW or lift (Figure 2). Standing time accuracy was calculated as the percentage of thigh‐derived standing time during the *device‐on* condition that the algorithm identified as occurring while using the device. Although the algorithm also considered walking time, we were not directly interested in accuracy of walking time, but rather step count accuracy, given the larger interest of quantifying cumulative plantar stress (which includes a term for the product of load/steps and steps/day [24]). If, for a given epoch, the algorithm indicated that the device was used while walking, then all thigh‐based steps during that epoch were considered to occur concurrently with the device. Step count accuracy with a device was calculated as the percentage of thigh‐derived steps during the *device‐on* condition that the algorithm identified as occurring while using the device.

Intraclass correlation coefficients (ICC; two‐way random effects model) were used to evaluate the level of agreement between actual (thigh‐monitor) activity (step‐count/standing time) during the *device‐on* condition and the amount of activity identified by the algorithm as occurring while using the device (RCW or lift, separately). ICC values > 0.9 indicated excellent agreement [27]. Error in an activity was calculated by first summing the amount of the activity identified as occurring with the device during: (i) the *device‐off* condition and (ii) the *device‐on* condition when the thigh‐monitor did not indicate activity; and then expressing the sum relative to the total amount of thigh‐derived activity. All ICC were performed in SPSS v23.0 (IBM, SPSS, Armonk, NY) with significance set at *α* ≤ 0.05.

### 2.6 Analyzing the 24‐h Community Data

While the laboratory data was used to train and validate our custom algorithm, the 24‐h community data provided insight into functionality of the algorithm for community‐based adherence monitoring. The algorithm, with optimized thresholds, was applied to this data to quantify step counts and standing time performed while using the RCW and lift monitors, that is, Algorithm‐derived Adherent (AA) Step Count (SC_AA_) and Algorithm‐derived Adherent Standing Time (ST_AA_), respectively (Figure 2). Similar metrics were then derived from the diaries. Specifically, diary entries were used to segment the 24‐h period into intervals where devices were donned or doffed. These intervals were then compared with corresponding time periods in the thigh‐monitor data. The total number of steps and standing time from the thigh monitor occurring when the devices were reported as donned defined the Diary‐derived Adherent (DA) Step Count (SC_DA_) and Diary‐derived Adherent Standing Time (ST_DA_).

Agreement between algorithm‐ and diary‐derived adherence measures for the RCW and for the lift were separately visualized using Bland Altman plots and ICC (two‐way random effects model) were used to quantify the agreement. As we could not monitor the degree of compliance with diary logging, we expected ICCs for the 24‐h data (algorithm vs. diary) to be lower than for the lab (algorithm vs. thigh during *device‐on*). Nonetheless, this served as an overall check of algorithm functionality.

## 3 Results

### 3.1 Initial Validation of Adherence Algorithm (In‐Lab Activities)

Of the 30 participants who completed the study, data from 27 were included in the in‐lab analysis; data from three participants were excluded due to errors in step and standing counts from the RCW and the lift monitors during the *device‐off* condition that were greater than two standard deviations above the mean. These cases likely reflected the investigator inadvertently moving the devices on multiple occasions after the devices were doffed by participants.

As anticipated, the use of the thigh as the reference during validation was strongly supported. During both the *device‐on* and *device‐off* conditions the thigh monitor had 100% ± 0% accuracy in classifying standing time and step counts in relation to those recorded by the investigator log. In addition, the VANE algorithm was considerably less accurate in identifying steps and standing from the device monitors. Accuracy of VANE algorithm to detect step counts and standing time from the RCW monitor during the *device‐on* condition was 71.0 ± 79.8% and 96.4 ± 3.18%, respectively; from the lift monitor, accuracies were 74.8 ± 79.8% and 88.3 ± 12.02%, respectively. These values were considerably improved with the custom algorithm. Accuracy of the custom algorithm to detect step counts and stand time while using the RCW during the *device‐on* condition was 100 ± 0% and 99.82 ± 0.65%, respectively; accuracies to detect events while using the lift were 99.25 ± 0.86% and 97.81 ± 4.40%, respectively. These accuracies corresponded to step counts and stand times that had excellent agreement with those from the thigh‐monitor (ICC > 0.991 and *p* < 0.01 for all comparisons). The algorithm also had minimal error. Step count errors were 1.00 ± 2.53% and 1.25 ± 2.75% for the RCW and lift monitors, respectively; for standing time they were 2.84 ± 3.10% and 3.00 ± 4.19%, respectively.

### 3.2 Analysis of 24‐h Community Data

**FIGURE 3:**
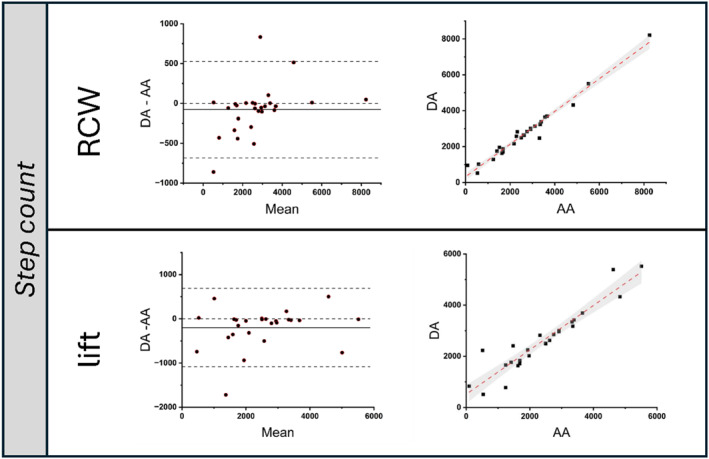
(Top left and bottom left) Bland‐Altman plots showing differences between the 24‐h diary‐ and algorithm‐derived adherent step counts (DA and AA, respectively) for the RCW (top) and for the lift (bottom). (top right and bottom right) Scatter plots of the diary‐versus. algorithm‐derived adherent steps with the RCW and with the lift. Grey area represents 95% confidence intervals about the best‐fit line, with the line of unity shown in dotted red for reference.

**FIGURE 4:**
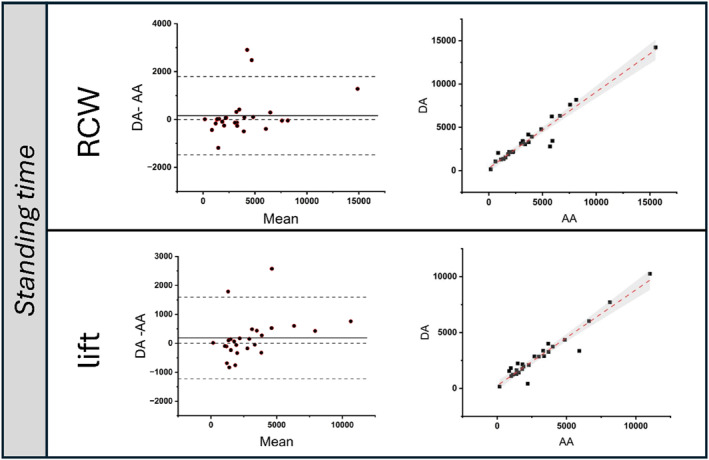
(Top left and bottom left) Bland‐Altman plots showing differences between the 24‐h diary‐ and algorithm‐derived adherent standing time (DA and AA, respectively) for the RCW (top) and for the lift (bottom). (top right and bottom right) Scatter plots of the diary‐versus. algorithm‐derived adherent standing time with the RCW or with the lift. Grey area represents 95% confidence intervals about the best‐fit line, with the line of unity shown in dotted red for reference.

For the analysis of 24‐h data, we included results from 27 participants. For three participants, we noted errors in step and standing counts with the RCW and the lift monitors' data that were greater than two standard deviations from the 24‐h diary logs. After further evaluation, the investigators did not have confidence in the diary entries and ultimately excluded these 3 participants from analysis. Among the remaining 27 participants, lift data from one participant was unavailable as their lift monitor fell off during monitoring. Overall, there was excellent agreement between algorithm‐ and diary‐derived adherent step counts with the devices (Figure 3; ICC > 0.965, *p* < 0.01) and between algorithm‐ and diary‐derived adherent standing time with the devices (Figure 4; ICC > 0.961, *p* < 0.01).

## 4 Discussion

We developed an algorithm to calculate adherent step counts and standing time using monitors integrated with a removable cast walker (RCW) and a contralateral shoe lift, to address the challenges of self‐reported adherence [21, 22, 28]. The algorithm was successfully validated using controlled in‐lab data. The true functionality of the algorithm was then evaluated based on 24 h of community‐wear compared with diary recordings of donning and doffing of the devices in the community. Excellent agreement between algorithm‐ and diary‐derived adherence (Figures 3 and 4) demonstrates the ability to provide an objective, reliable alternative to self‐reported community‐based adherence.

These findings build upon prior work [22, 26] by assessing adherence during standing and walking, and by concurrently assessing adherence with an RCW and with a contralateral shoe‐lift. Although the International Working Group on the Diabetic Foot (IWGDF) recently emphasized the potential benefits of providing shoe‐lifts to mitigate induced limb length discrepancy [8], prior studies have not objectively monitored patients' use of shoe‐lifts. Monitoring shoe‐lift usage may provide a more complete picture of behaviors that can influence adherence and healing during offloading regimes. Indeed, shoe‐lifts have been shown to minimize adverse effects of RCW wear—including discomfort, joint pain and instability during gait [25, 29, 30]—that may result from the limb‐length discrepancy induced by offloading modalities. Evidence also suggests that shoe‐lifts can reduce the perceived effort of walking with an offloading device [31]. Thus, use of a contralateral shoe‐lift is anticipated to improve RCW usability—the extent to which the RCW can be used by patients to achieve wound healing with effectiveness, efficiency, and satisfaction [32]. Accordingly, consistent use of a contralateral shoe‐lift may optimize user experiences during offloading regimes, leading to greater use (adherence) of the RCW itself.

The ability to objectively monitor walking and standing adherence not only allows quantification of offloading adherence as an outcome in research studies but also provides opportunities to enhance interventions and offloading practices. Real‐time, reliable quantification of non‐adherent behaviors could help inform individualized feedback during interventions [33, 34]. Such an approach has shown promise in a preliminary investigation of the use of monitoring offloading adherence within a behavioral intervention [34]. However, it should be noted that the methods used in that interventional investigation had several limitations relative to the present study's. Two of those include limiting adherence determinations of weightbearing activity to episodes of walking and only capturing adherence to the offloading device on the wounded foot (no assessment of contralateral footwear).

In addition to assisting patients directly, objective OA data can support clinicians in differentiating between pathophysiological and behavioral causes of non‐healing DFUs, promoting more targeted, patient‐specific management strategies. For instance, poor ulcer healing despite high adherence detected by the system may indicate the need to focus on alternative medical treatment plans. Conversely, low healing in combination with low adherence might prompt focused counseling and education to change behavior. Overall, given the association between adherence and DFU healing [10], the ability to monitor and enhance OA through objective means could translate into measurable reductions in complications related to non‐healing wounds.

While the current method achieved high accuracy in quantifying standing time and walking with the devices, several opportunities remain for refinement. For example, the ability to automatically detect specific tasks such as stair ambulation or inclined walking that can modify plantar pressure profiles [35] could improve the ecological validity of future algorithms designed to quantify the total amount of stress imparted to the foot during daily activities (cumulative plantar tissue stress [24]).

Several limitations should be noted. First, the study included healthy adults without neuropathy or active DFUs, which may limit the generalizability of findings to clinical populations. Future work may consider replicating validation in patients with diabetes and neuropathy to account for altered gait mechanics and step‐to‐step variability. Additionally, the 24‐h monitoring window, though adequate for validation, may not fully capture day‐to‐day variability in OA behaviors. Longer monitoring durations (3–4 weeks) would provide greater insight into OA patterns and the stability of the algorithm's accuracy over time.

## 5 Conclusion

The study developed and validated an algorithm that included data from three accelerometers to objectively and accurately detect walking and standing activities performed while using an RCW and a contralateral shoe‐lift, as a means to quantify community‐based adherence with these devices. The system demonstrated very strong agreement with both in‐lab observations and user diaries, supporting its use for real‐world monitoring of offloading behavior. This method offers the opportunity for enhanced investigation of offloading adherence in clinical trials. It may also provide a foundation for enhancing clinical decision‐making in DFU management via future integration into standard clinical practice.

## Author Contributions

**Sai V. Yalla:** conceptualization, formal analysis, software, supervision, visualization, writing – original draft, writing – review and editing. **Sanam N. Jhaveri:** data curation, visualization, writing – original draft, writing – review and editing. **Nahir S. Negron‐Fernandez:** data curation, visualization, writing – review and editing. **Ryan T. Crews:** conceptualization, project administration, supervision, writing – review and editing, funding acquisition. **Noah J. Rosenblatt:** conceptualization, formal analysis, project administration, writing – original draft, writing – review and editing, funding acquisition.

## Funding

This work was partially supported by the National Institute of Diabetes and Digestive and Kidney Diseases under awards R01DK131303 and T35DK074390.

## Disclosure

The authors have nothing to report.

## Ethics Statement

This study that was approved by the Rosalind Franklin University IRB and all individuals provided written informed consent to participate.

## Conflicts of Interest

The authors declare no conflicts of interest.

## Data Availability

The data that support the findings of this study are available from the corresponding author upon reasonable request.
